# Rare clinical presentations of pleomorphic xanthoastrocytoma with a high proliferative index: Two case reports

**DOI:** 10.1097/MD.0000000000018880

**Published:** 2020-01-17

**Authors:** Masaya Nagaishi, Ryuta Nakae, Yoshiko Fujii, Yuki Inoue, Yoshiki Sugiura, Issei Takano, Yoshihiro Tanaka, Kensuke Suzuki

**Affiliations:** Department of Neurosurgery, Dokkyo Medical University Saitama Medical Center, 2-1-50 Minami-Koshigaya, Koshigaya-shi, Saitama, Japan.

**Keywords:** 5-aminolevulinic acid, glioma, multicentric, pineal, pleomorphic xanthoastrocytoma

## Abstract

**Introduction:**

Pleomorphic xanthoastrocytomas (PXA) are rare, typically benign, slow-growing tumors that commonly occur in the cerebral hemispheres. We describe two cases of clinically aggressive PXA with uncommon locations; one was in the tectal plate, and the other had simultaneous multicentric lesions.

**Patient Concerns:**

The both cases presented with severe headache with no significant past medical history.

**Diagnosis:**

PXA World Health Organization grade II were histopathologically diagnosed from surgically resected specimens, and immunohistochemical and sequence analysis revealed a high Ki-67 proliferative index and BRAF V600E mutation in both the cases.

**Interventions:**

The first case presented with multicentric lesions and underwent partial resection, whereas the second case presented with a tectal plate tumor that was managed by gross total surgical resection. Strong 5-aminolevulinic acid (5-ALA)-induced fluorescence was observed in both the lesions. Postoperative radiotherapy plus concomitant and adjuvant temozolomide was administered to both the patients.

**Outcomes:**

Despite completing adjuvant chemo-radiotherapy, both the patients had local tumor recurrence at 2 and 5 months after the operation, respectively.

**Conclusion:**

The progressive clinical courses in our cases suggest that additional postoperative therapy should be considered during the treatment of PXA with a high Ki67 index, and that temozolomide with radiotherapy, followed by temozolomide maintenance therapy, may not prevent recurrence in such tumors. Importantly, our experience implies that unlike other subtypes of low grade gliomas, 5-ALA fluorescence is useful for intraoperative visualization of PXA.

## 1 Introduction

Pleomorphic xanthoastrocytoma (PXA) are a rare, low-grade glioma subtype that account for less than 1% and are characterized by a unique histopathological pattern.^[1]^ They most commonly affect children and young adults, typically originate from the supratentorial compartment, and are most frequently observed in the temporal lobe with very a few lesions in other areas.

Very few reports have described simultaneous multicentric PXA.^[2,3]^ PXAs are associated with a favorable prognosis, and the current 5-year recurrence-free survival rate after surgical resection is 70.9%. In contrast, the 5-year recurrence-free survival rate is significantly shorter (48.9%) in PXAs with anaplastic features; this type of lesion is now classified as anaplastic PXA (Grade III) in the 2016 World Health Organization (WHO) classification of Tumors of the Central Nervous System.^[4]^ Anaplastic PXA is defined as PXA with a mitotic index ≥5 per 10 high-power fields and/or necrosis.^[5]^ Although proliferative activity does not affect tumor grading in PXA, some authors have emphasized the use of the Ki-67 index as a prognostic factor.^[6,7]^ Here, we describe two cases of clinically uncommon PXA with aggressive clinical courses, both with a high Ki67 index, wherein one patient presented with a multicentric lesion and the other, a tectal plate tumor.

## 2 Case presentations

### 2.1 Case 1

**Figure 1:**
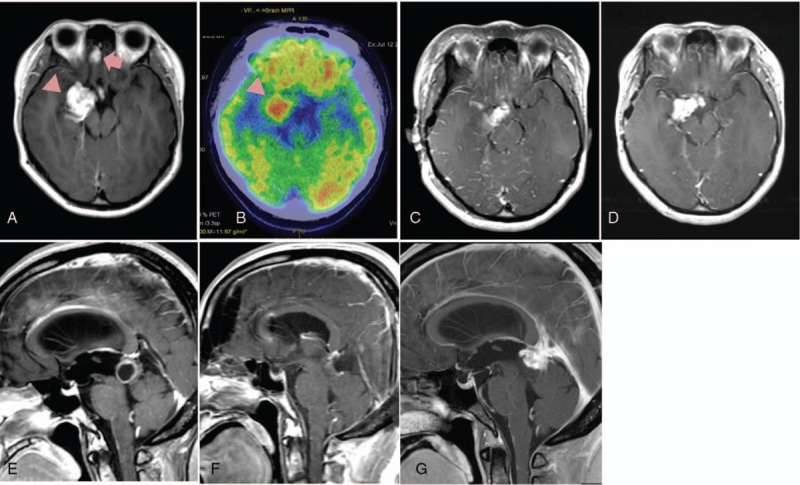
PET and gadolinium-enhanced MR imaging in case 1 (A–D) and case 2 (E–G). Case 1: Preoperative MRI shows two enhancing lesions in the frontal (A, arrow) and temporal lobes (A, arrow head), and PET imaging demonstrates high fluorodeoxyglucose (^18^F) uptake in the temporal lesion (B, arrow head). Partial removal of the tumors was confirmed by postoperative MRI (C), and MRI after adjuvant therapy revealed regrowth of the residual tumor (D). Case 2: Preoperative MRI shows a cystic tumor in the tectal plate (E). Complete resection of the tumor was confirmed by postoperative MRI (F). MRI after adjuvant therapy indicated local recurrence of the tumor (G).

A 19-year-old female presented with severe headache. A neurological examination revealed no significant findings. Gadolinium-enhanced magnetic resonance imaging (MRI) revealed a distinct and enhanced mass lesion in the medial temporal lobe and the right orbital gyrus, both of which showed slight growth on a follow-up MRI at 1-month after initial presentation (Fig. 1A). Neither cyst formation nor calcification was detected in the mass lesions. Positron emission tomography (PET) revealed high ^18^F-fluorodeoxyglucose (FDG) uptake in both the lesions (Fig. 1B). She underwent right front-temporal craniotomy with tumor resection, and strong 5-aminolevulinic acid (5-ALA)-induced fluorescence was detected intraoperatively. The tumor in the right orbital gyrus was totally resected, but that in the temporal lobe could only be partially resected because of tumor invasion of the cerebral penetrating brunch (Fig. 1C). While the temporal lesion was an intra-axial tumor, the tumor of the right orbital gyrus was located in the subpial space. The residual tumor was treated with radiotherapy (54Gy/30fr) and concomitant (75 mg/m^2^/day, 7 days/week for 6 weeks) and adjuvant (150 mg/m^2^/day, 5 days/month) temozolomide following surgery. The reduction in size of residual tumor was not observed by adjuvant therapy. She remained stable without deterioration in her clinical symptoms, however, MRI at 3 months after the operation revealed tumor regrowth (Fig. 1D).

### 2.2 Case 2

A 47-year-old woman presented with progressive headaches and disturbance of consciousness. Neurological examination confirmed disorientation and recent memory impairment. Computerized tomography revealed hydrocephalus caused by a single cystic tumor with calcification in the tectal plate, and the mural nodule exhibited gadolinium enhancement on T1-weighted MRI (Fig. 1E). She underwent surgery for a suspected low grade glioma, and the tumor was totally resected via a right occipital transtentorial approach (Fig. 1F). Intraoperative findings demonstrated a reddish purple tumor with strong 5-ALA-induced fluorescence. She was administered radiotherapy (54Gy/30fr) with concomitant (75 mg/m^2^/day, 7 days/week for 6 weeks) and adjuvant (150 mg/m^2^/day, 5 days/month) temozolomide following the surgery, despite which tumor recurrence was detected on follow-up MRI at 5 months after the operation (Fig. 1G). She was admitted to hospital with progressively worsening level of consciousness due to tumor invasion, and began treatment with bevacizumab (10 mg/kg every 2 weeks).

### 2.3 Pathological findings

**Figure 2:**
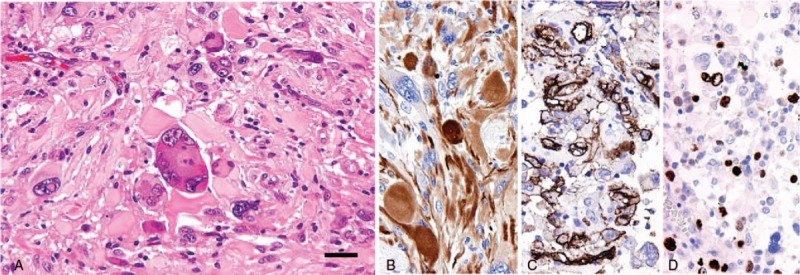
Micrographic findings in case 2. Micrography revealed a highly cellular tumor composed of bizarre glial cells with prominent nuclear atypia and xanthomatous cells (HE, A). Tumor cells showed high expression levels of GFAP (B), CD34 (C), and Ki-67 (D). The scale bar in (A) indicates 50 μm for A–D.

Microscopic analysis of the resected tumors from both the patients revealed highly cellular tissue comprising atypical astrocytic tumor cells, including bizarre multinucleated cells and xanthomatous cells (Fig. 2A). Mitosis was rarely observed, and both the cases were devoid of microvascular proliferation and necrosis. Immunohistochemistry demonstrated GFAP and CD34 expression in both the cases (Fig. 2B, C). The Ki-67 labeling index (LI) values for cases 1 and 2 were 20% and 8.7%, respectively, (Fig. 2D). Sequence analysis revealed *BRAF V600E* mutation in both the cases, but no mutations in *IDH1/2* genes or the *TERT* promoter. Quantitative reverse-transcription PCR confirmed the absence of *KIAA1549-BRAF* fusion. Thus, both the cases were histopathologically diagnosed as PXA WHO grade II. Patients have provided informed consent for publication of the cases.

## 3 Discussion

Few cases of PXA with simultaneous multicentric lesions have been previously described. In the first case of the present report, intraoperative finding of a subpial mass in the orbital gyrus was consistent with cerebrospinal fluid dissemination. PXA commonly displays leptomeningeal dissemination or malignant transformation during long-term follow-up but rarely exhibits dissemination at initial presentation. Our second patient had an extremely rare PXA tumor location, the tectal plate. Tectal gliomas are relatively noninvasive and slow-growing tumors that are have a less aggressive clinical course.^[8]^ Histopathologically, tectal gliomas are predominantly low grade gliomas, and pilocytic astrocytomas are the most frequent type.^[9]^ Nonetheless, a few cases of pineal or tectal region PXA have been reported,^[10,11]^ and as most of these tumors are involved in both the pineal and the tectal region, the site of tumor origin remains unclear. In our case, preoperative MRI and intraoperative findings confirmed tumor location as distal to the normal pineal body, with the tumor predicating as a tectal PXA.

It is difficult to assess the malignancy of PXA based on histopathological appearance alone, even though malignant PXA is diagnosed if necrosis and a high mitotic index are observed. Previous reports have indicated that a high Ki67 LI index and *BRAF* status also correlate with malignancy in PXA.^[7]^ The presence of *BRAF V600E* mutation, which is observed in 65% of PXA cases, is a predictor of favorable prognosis.^[4,12,13]^ Tabouret et al. have reported that the *BRAF V600E* mutation is associated with lower Ki67 LI values;^[13]^ however, both the cases described here exhibited a progressive clinical course despite the presence of the *BRAF V600E* mutation and absence of high mitotic activity, necrosis, or microvascular proliferation. Thus, our experience suggests that additional therapy following curative resection should considered during treatment of PXA with high Ki67 LI values.

Recurrence and overall survival of PXA with anaplastic features are significantly lower than those of PXA without anaplastic features,^[4,14]^ and additional chemo/radiotherapy is commonly recommended in patients with anaplastic PXA. However, the paucity of clinical studies hampers the selection of appropriate postoperative therapy, and the benefits of radiotherapy and chemotherapy with temozolomide or vemurafenib remain controversial. Importantly, both our patients did not respond to this combination of radiotherapy and chemotherapy.

High FDG uptake is commonly observed in recurrent and anaplastic PXA, but is rarely seen in typical PXA.^[15]^ However, we assumed that the hypermetabolic state observed in case 1 was associated with tumor aggressiveness, and FDG-PET may be a useful preoperative assessment tool for tumor aggressiveness in PXA.

The intraoperative use of 5-ALA fluorescence for guidance is an established practice for the visualization of gliomas. Positive fluorescence is generally observed in high grade gliomas, ependymoma, and medulloblastoma, but is less frequently seen in WHO grade II gliomas, including astrocytomas and oligodendrogliomas (15.8%), and is rarely seen in pilocytic astrocytomas and gangliogliomas.^[16,17]^ Only few cases have reported the use of 5-ALA fluorescence for PXA and Jaber et al have also indicated that ^18^F-FET PET uptake can predict 5-ALA-induced fluorescence.^[16]^ Interestingly, contrary to other WHO grade II gliomas, all the reported cases of PXA, including those reported here, displayed 5-ALA enhancement.^[18,19]^ Therefore, we propose the use of intraoperative 5-ALA fluorescence guidance in all the patients with PXA.

## 4 Conclusion

We describe 2 cases of PXA with unusual clinical presentations and the case reports suggest that high Ki-67 LI in PXA can be used as a predictive marker of an adverse clinical course and that a combination of radiotherapy and chemotherapy with temozolomide may not be beneficial for patients with PXA with high Ki-67 LI values. Conversely, 5-ALA fluorescence is potentially beneficial for intraoperative tumor visualization in PXA.

## Acknowledgments

We wish to thank Kyoko Tsujikado (Koshigaya Division of Clinical Research, Institute for Medical Science) for providing technical support and Enago for the English language review. We declare that we have no conflict of interest.

## Author contributions

**Conceptualization:** Issei Takano.

**Data curation:** Yoshiko Fujii, Yuki Inoue, Yoshiki Sugiura.

**Supervision:** Ryuta Nakae, Kensuke Suzuki.

**Visualization:** Yoshihiro Tanaka.

**Writing – original draft:** Masaya Nagaishi.

## References

[1] Rao AA Laack NN Giannini C Pleomorphic xanthoastrocytoma in children and adolescents. Pediatr Blood Cancer 2010;55:290–4. 20582976 10.1002/pbc.22490

[2] McNatt SA Gonzalez-Gomez I Nelson MD Synchronous multicentric pleomorphic xanthoastrocytoma: case report. Neurosurgery 2005;57:E191 discussion E91. 15987556 10.1227/01.neu.0000164174.07281.f9

[3] Saikali S Le Strat A Heckly A Multicentric pleomorphic xanthoastrocytoma in a patient with neurofibromatosis type 1. Case report and review of the literature. J Neurosurg 2005;102:376–81. 15739569 10.3171/jns.2005.102.2.0376

[4] Ida CM Rodriguez FJ Burger PC Pleomorphic xanthoastrocytoma: natural history and long-term follow-up. Brain Pathol 2015;25:575–86. 25318587 10.1111/bpa.12217 PMC4400218

[5] Louis DN Perry A Reifenberger G The 2016 World Health Organization Classification of Tumors of the Central Nervous System: a summary. Acta Neuropathol 2016;131:803–20. 27157931 10.1007/s00401-016-1545-1

[6] Hirose T Ishizawa K Sugiyama K Pleomorphic xanthoastrocytoma: a comparative pathological study between conventional and anaplastic types. Histopathology 2008;52:183–93. 18184267 10.1111/j.1365-2559.2007.02926.x

[7] Sugita Y Shigemori M Okamoto K Clinicopathological study of pleomorphic xanthoastrocytoma: correlation between histological features and prognosis. Pathol Int 2000;50:703–8. 11012983 10.1046/j.1440-1827.2000.01104.x

[8] Igboechi C Vaddiparti A Sorenson EP Tectal plate gliomas: a review. Childs Nerv Syst 2013;29:1827–33. 23612874 10.1007/s00381-013-2110-z

[9] Ternier J Wray A Puget S Tectal plate lesions in children. J Neurosurg 2006;104:369–76. 10.3171/ped.2006.104.6.369 16776370

[10] Katayama K Asano K Shimamura N Case of pleomorphic xanthoastrocytoma with anaplastic features in the pineal gland. Brain Tumor Pathol 2013;30:242–6. 23460303 10.1007/s10014-013-0137-1

[11] Suzuki Y Akiyama Y Kimura Y Pleomorphic xanthoastrocytoma with anaplastic features in the tectal region in a young adult patient: a case report. World Neurosurg 2016;94: 580.e11-80.e15. 10.1016/j.wneu.2016.07.110 27521728

[12] Schmidt Y Kleinschmidt-DeMasters BK Aisner DL Anaplastic PXA in adults: case series with clinicopathologic and molecular features. J Neurooncol 2013;111:59–69. 23096133 10.1007/s11060-012-0991-4 PMC4617340

[13] Tabouret E Bequet C Denicolai E BRAF mutation and anaplasia may be predictive factors of progression-free survival in adult pleomorphic xanthoastrocytoma. Eur J Surg Oncol 2015;41:1685–90. 26454767 10.1016/j.ejso.2015.09.012

[14] Perkins SM Mitra N Fei W Patterns of care and outcomes of patients with pleomorphic xanthoastrocytoma: a SEER analysis. J Neurooncol 2012;110:99–104. 22843450 10.1007/s11060-012-0939-8

[15] Etzl MM Jr Kaplan AM Moss SD Positron emission tomography in three children with pleomorphic xanthoastrocytoma. J Child Neurol 2002;17:522–7. 12269732 10.1177/088307380201700709

[16] Jaber M Wolfer J Ewelt C The Value of 5-Aminolevulinic acid in low-grade gliomas and high-grade gliomas lacking glioblastoma imaging features: an analysis based on fluorescence, magnetic resonance imaging, 18F-Fluoroethyl tyrosine positron emission tomography, and tumor molecular factors. Neurosurgery 2016;78:401–11. discussion 11. 26366972 10.1227/NEU.0000000000001020 PMC4747980

[17] Preuss M Renner C Krupp W The use of 5-aminolevulinic acid fluorescence guidance in resection of pediatric brain tumors. Childs Nerv Syst 2013;29:1263–7. 23708867 10.1007/s00381-013-2159-8

[18] Ruge JR Liu J Use of 5-aminolevulinic acid for visualization and resection of a benign pediatric brain tumor. J Neurosurg Pediatr 2009;4:484–6. 19877785 10.3171/2009.6.PEDS08428

[19] Valdes PA Jacobs V Harris BT Quantitative fluorescence using 5-aminolevulinic acid-induced protoporphyrin IX biomarker as a surgical adjunct in low-grade glioma surgery. J Neurosurg 2015;123:771–80. 26140489 10.3171/2014.12.JNS14391 PMC4646619

